# Ochratoxin A induces mitochondrial dysfunction, oxidative stress, and apoptosis of retinal ganglion cells (RGCs), leading to retinal damage in mice

**DOI:** 10.1007/s10792-024-03032-w

**Published:** 2024-02-13

**Authors:** Miao Fu, Yuanyuan Chen, Anhuai Yang

**Affiliations:** Department of Ophthalmology, Renmin Hospital of Wuhan University, Wuhan, Hubei China

**Keywords:** Ochratoxin A, Retinal ganglion cells, Oxidative stress, Apoptosis, Mitochondrial

## Abstract

**Purpose:**

Ochratoxin A (OTA) contamination of food and feed is a serious problem worldwide. OTA is considered a carcinogen and immunotoxic, nephrotoxic, and neurotoxic mycotoxin. The present study aims to determine the toxic effects of OTA on retinal ganglion cells (RGCs) and assess the resulting impairment of retinal function in mice.

**Methods:**

RGC-5 cells were exposed to OTA (100 and 200 μg/L) for 3 days, and the mice were fed OTA-contain (100 and 200 μg/kg) diets for 4 weeks. Antioxidant indices were detected by spectrophotometer. The apoptosis of RGC-5 cells was determined by flow cytometry. Mitochondrial morphology and mitochondrial membrane potential were detected by immunofluorescence. RGC survival was determined by immunofluorescence staining with Brn3a. Flash electroretinography (ERG) was conducted to assess visual function.

**Results:**

The oxidative-antioxidant balance suggested that OTA-induced severe oxidative stress, including increased reactive oxygen species (ROS) and malondialdehyde (MDA) levels in the OTA-exposed RGC-5 cells, and the reduced activity of superoxide dismutase (SOD) and glutathione-S-transferase (GST) in the OTA exposed group. Furthermore, OTA exposure led to remarkable apoptosis in RGC-5 cells. The mitochondrial detection showed that OTA caused significant mitochondrial membrane potential reduction and mitochondrial fragmentation, which may be the cause of apoptosis of RGC-5 cells. Additionally, in vivo experiments demonstrated that OTA resulted in significant death of RGCs and subsequent retinal dysfunction in mice.

**Conclusion:**

Ochratoxin A induces mitochondrial dysfunction, oxidative stress, and RGCs death in mice.

## Introduction

Vision is an important sense for humans, and visual impairment will produce a series of barriers in daily life. The retina is responsible for light transduction and for preprocessing this information with an intraretinal circuitry. Retinal ganglion cells (RGCs) are the only projecting neurons of the retina, forming the optic nerve to transmit visual information to the brain [1]. The progressive degeneration and death of RGC could cause irreversible vision decline and blindness, such as glaucoma, which affects more than 70 million people worldwide [2]. It is generally believed that RGC degeneration is involved in ischemia, oxidative stress, neurotoxicity, inflammation, and neurotrophic factor deprivation [3]. The retina has a blood–riboflavin–retinal barrier, which protects the retinal cells from a wide-range of substances or microorganisms [3]. However, several agents, such as qui-no-lines, phenothiazines, antiretroviral drugs, and some toxic substances, can reach the retina and influence RGC’s function and survival [4, 5].

Ochratoxin is a mycotoxin produced by *Aspergillus ochraceus* and several fungi belonging to the Penicillium genus. Ochratoxin has three main analogs: ochratoxin A (OTA), ochratoxin B (OTB), and ochratoxin C (OTC). Among these different types of ochratoxins, OTA is the most toxic, is serious contaminants of our food, crops, water, and animals feed [6]. OTA can be absorbed and distributed to various tissues and organs, causing multiorgan toxicity in humans and animals, such as nephrotoxicity, hepatotoxicity, embryotoxicity, immunotoxicity, developmental toxicity, carcinogenicity, and genotoxic apoptosis [7, 8]. Previous studies have confirmed that OTA exposure could cause organ and tissue damages in kidney, liver, and gastrointestinal tract [9]. And it has also been verified to cause extensive neurotoxicity due to its ability to cross the blood–brain barrier and reach the brain and nervous system [10].

In this study, we aimed to characterize the potential toxic effects of OTA on RGCs and its mechanism. In vitro experiments have shown that OTA can cause oxidative stress, apoptosis, mitochondrial membrane potential reduction, and mitochondrial fragmentation in RGC-5 cells. The RGCs survival and retinal function of mice exposed to OTA were assessed in vivo. This study will most likely provide a fresh perspective to understand the toxicological effects of OTA.

## Materials and methods

### Cell culture and animal husbandry

The RGC-5 cells were purchased from iCell Bioscience Inc. (Shanghai, China) and cultured in RPMI-1640 medium supplemented with 10% fetal bovine serum (FBS, Gibco-BRL, Grand Island, USA), penicillin (100 IU/mL), and streptomycin (0.1 mg/mL) (Meiji Seika, Tokyo, Japan). Cells were grown in a humidified atmosphere with 5% CO_2_ at 37 °C. One passage was performed at a ratio of 1:4 every 3–4 days along with trypsin digestion.

Wild-type adult male C57BL/6 J mice (8 weeks old) purchased from the Animal Laboratory Center of Wuhan University. The mice raised in temperature and humidity-controlled rooms on a 12-h light/12-h dark cycle. All experiments were performed according to the National Institutes of Health guidelines for the Animal Care and Use and the ARVO Statement for the Use of Animals in Ophthalmic and Vision Research.

### Exposure of OTA to RGC-5 cells and mice

OTA (purity > 98%) was purchased from Sigma–Aldrich (Sigma–Aldrich, St. Louis, MI, USA). OTA solution (50,000 mg/L) as a stock solution was prepared with dimethyl sulfoxide (DMSO; Aladdin, China) for further use. RGC-5 cells were seeded into a 12-well plate and incubated at 37 °C until they reached confluence, and concentrations of 100 and 200 μg/L OTA or up to 0.1% DMSO were added to wells in triplicate for up to 3 days. To maintain the actual concentrations of OTA, during exposure period, the cell medium containing OTA was exchanged by daily. The mice were divided into three groups and exposed to OTA for 4 weeks, the concentrations of 0, 100 and 200 μg/kg OTA were added to the feeds of mice (0, 0.023, and 0.046 µg/g per body weight). There were three replicates in each group.

### Antioxidant Indices

The sampled RGC-5 cells were added in sterile phosphate-buffered saline (PBS) at a ratio of 1:9 (*w/v*) and homogenized with a glass homogenizer at 4 °C. The homogenate was centrifuged at 5000* g* for 20 min at 4 °C. The supernatant was separated and used to test the superoxide dismutase (SOD) activity, glutathione-S-transferase (GST) levels, malondialdehyde (MDA), and reactive oxygen species (ROS) levels using appropriate kits according to the manufacturer’s instructions (Jiancheng, Nanjing, China).

### Cell apoptosis detection

The apoptosis of RGC-5 cells induced by OTA were detected by Annexin V-FITC/PI Apoptosis Detection Kit Anne (YEASEN, Shanghai, China). The sampled RGC-5 cells were collected and rinsed twice with pre-cooled sterile PBS. Then, the RGC-5 cells were centrifuged at 3000* g* for 10 min at 4 °C and resuspended in 1 × binding buffer, and the FITC-labeled annexin V (5 μL) and propidium iodide (10 μL) were added. Subsequently, the mixtures were incubated for 15 min at room temperature in the dark and detected by flow cytometry (Becton Dickinson, CA, USA).

### Mitochondrial detection

Mitochondrial morphology: The sampled RGC-5 cells were incubated with a mitochondrial marker specific primary antibody (TOMM20, Abcam, Cambridge, UK) at 4 °C overnight. The cells were further incubated with secondary antibody (Alexa Fluor 488-labeled, Invitrogen, CA, USA) and DAPI. Images were visualized using an upright fluorescence microscope (Olympus, Tokyo, Japan).

Mitochondrial membrane potential: The mitochondrial membrane potential of RGC-5 cells exposed to OTA was assessed by using the fluorescent lipophilic cationic dye JC-1 and the Mitochondrial Membrane Potential Assay Kit (C2006, Beyotime, Hangzhou, China). The RGC-5 cells were treated with 5 μg/mL JC-1 dye and incubated for 30 min in the dark at 37 °C. After washing the cells three times with PBS, fluorescence images were acquired using the fluorescence microscope (Olympus, Tokyo, Japan).

### Retinal flat-mount immunostaining

Four weeks after exposed to OTA, the mice were anesthetized with 1% pentobarbital sodium, and then the eyeballs were extracted and further fixed in 4% paraformaldehyde for 1 h. The retinas were isolated and blocked overnight with 5% BSA in PBST (0.5% Triton X-100 in PBS) at 4 °C, followed by incubation with Brn3a antibody (Abcam, Cambridge, UK) for 48 h at 4 °C and finally by incubation with the secondary antibody (Alexa Fluor 594, Abcam, Cambridge, UK) overnight. After washing with PBS, the retinas were carefully incised to four radial semblances and flattened on a glass slide. The flat-mounts images were captured by a fluorescence microscope (BX51, Olympus, Tokyo, Japan). Two areas of each retinal petal located at 0.8–1.2 mm and 1.8–2.2 mm distance from the optic nerve head were counted [2]. The rates of survival RGCs were calculated by ImageJ software (National Institutes of Health, Bethesda, MD, USA).

### Scotopic flash electroretinography

Electroretinography (ERG) testing was conducted using the RetiMINER-C system (IRC Medical Equipment Co., Ltd, Chongqing, China) after mice exposed to OTA for 4 weeks, as previously described [11]. All mice were dark-acclimated overnight before ERG recording. After anesthetized, the mice pupils were dilated with compound tropicamide ophthalmic solution by topical application. Two active platinum electrodes were smoothly placed on the corneal surface. And placing a reference electrode subdermally between the two ears, and a ground electrode in the midline of the tail. Sterile saline solution was used to keep the eyes moist during the ERG recording and to improve the electrical contact between the electrode and the cornea. For the scotopic flash ERG, a series of light intensities at 0.01, 0.03, 0.1, 0.3, 1.0, and 3.0 cd s/m^2^ were recorded. Amplitudes of the A-wave and B-wave at 1.0 cd s/m^2^ were recorded for analysis.

### Statistical analysis

For the experiments, the Tukey test of one-way analysis of variance (ANOVA) was used for determining significance (SPSS 18.0, Chicago, USA) and expressed as the arithmetic mean ± standard deviation (SD). *p*-values < 0.05 indicating statistical significance.

## Results

### Oxidative stress in RGC-5 cells exposed to OTA

**Fig. 1:**
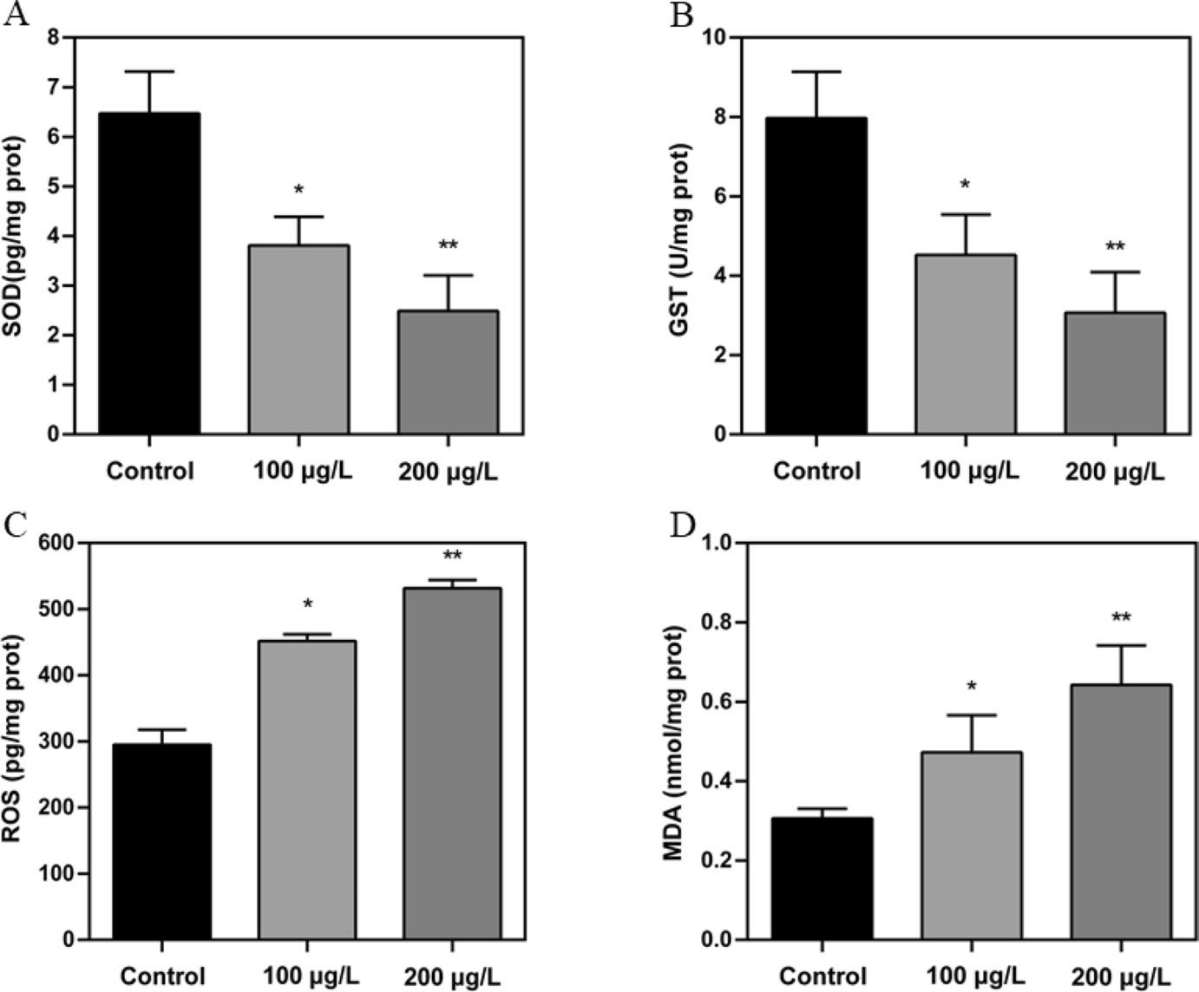
Oxidative stress in RGC-5 cells induced by OTA. **a** Superoxide dismutase (SOD). **b** Glutathione-S-transferase (GST).**c** Reactive oxygen species (ROS). **d** Malondialdehyde (MDA). Data are presented as mean ± standard deviation (*p < 0.05, **p < 0.01)

The results presented in Fig. 1 demonstrated a significant reduction in the levels of SOD and GST, as well as an increase in ROS and MDA levels in RGC-5 cells exposed to 100 and 200 μg/L OTA compared to the control group (*p* < 0.05). Specifically, the SOD and GST levels in cells exposed to 100 μg/L OTA were 58.8 and 56.7% of those in the control group, respectively, while ROS and MDA levels were 52.9 and 56.1% higher than in the control group. Similarly, in cells exposed to 200 μg/L OTA, the SOD and GST levels were 40.1 and 39.5% of those in the control group respectively, while ROS and MDA levels were 79.7 and 113% higher than in the control group.

### OTA-induced apoptosis of RGC-5 cells

**Fig. 2:**
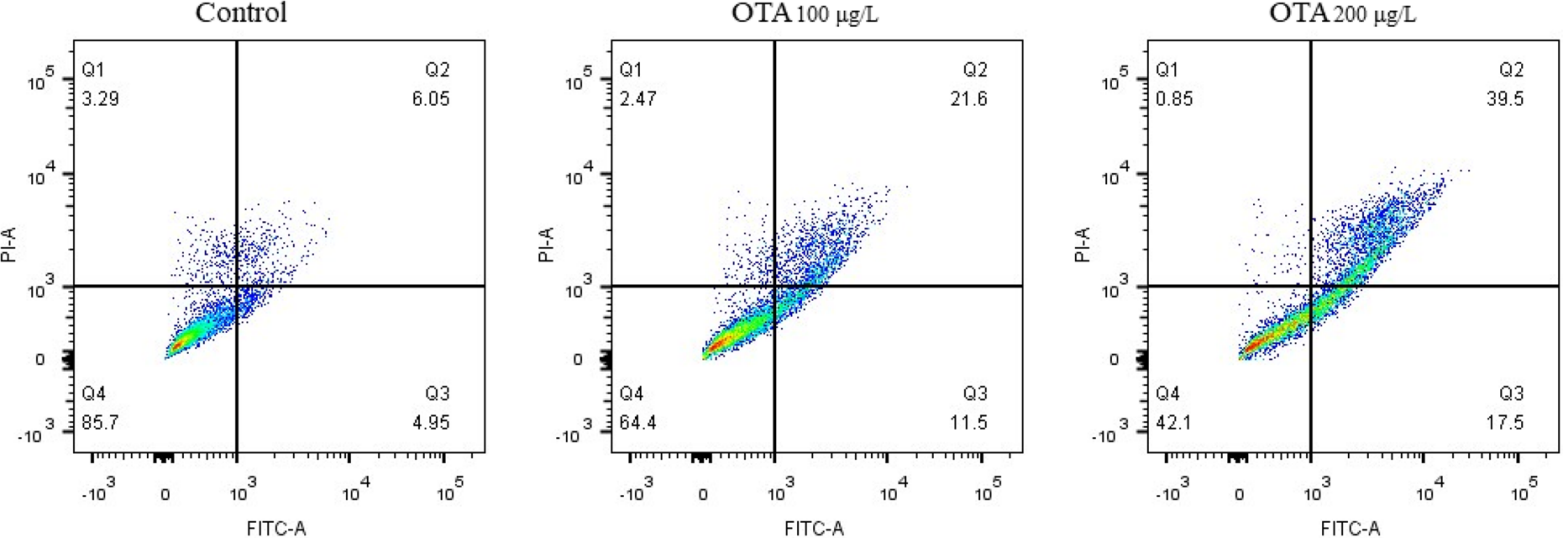
The apoptosis of RGC-5 cells exposed to OTA (0, 100, and 200 μg/L) were detected with Annexin V stain and flow cytometer

An Annexin V-FITC/PI assay was used to measure apoptosis in RGC-5 cells exposed to OTA. Flow cytometry was employed to analyze the results, and the Fig. 2 clearly indicated that OTA significantly elevated the proportion of apoptotic cells. Furthermore, the proportion of apoptotic cells increased in a concentration-dependent manner, suggesting that OTA toxicity is dose-dependent.

### Mitochondrial morphology

**Fig. 3:**
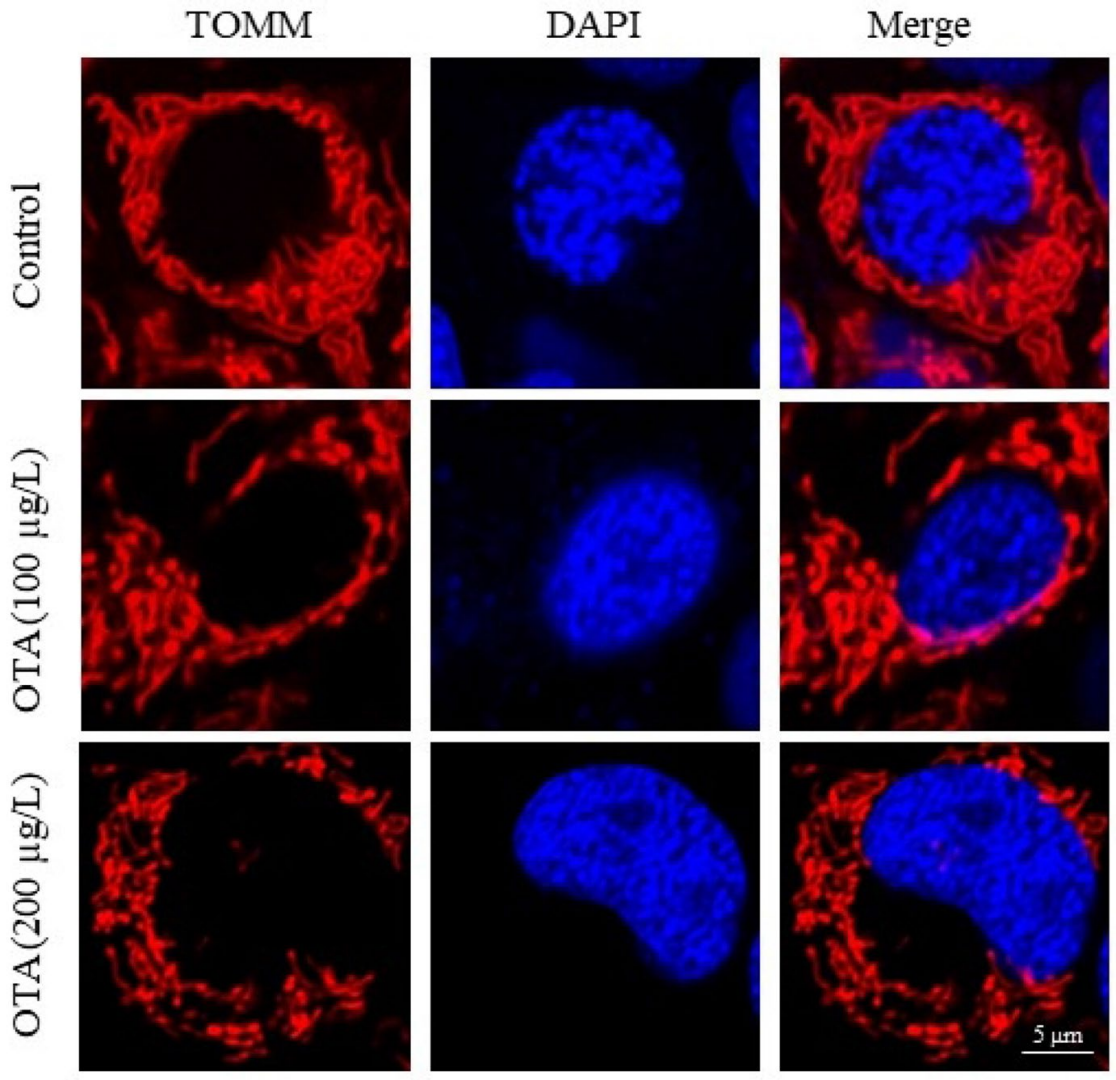
OTA-induced mitochondrial fragmentation in RGC-5 cells observed by fluorescence microscope. RGC-5 cells were treated with DAPI (blue) and TOMM20 (red). Blue represents nucleus, red represents mitochondria

Using fluorescence microscopy, we observed and compared the changes in mitochondrial morphology between RGC-5 cells exposed to OTA for 3 days and normal cells. We found that compared to normal cells, exposure to OTA caused a decrease in tubular mitochondria and spherical mitochondria became the predominant morphology in RGC-5 cells (Fig. 3).

### Mitochondrial membrane potential

**Fig. 4:**
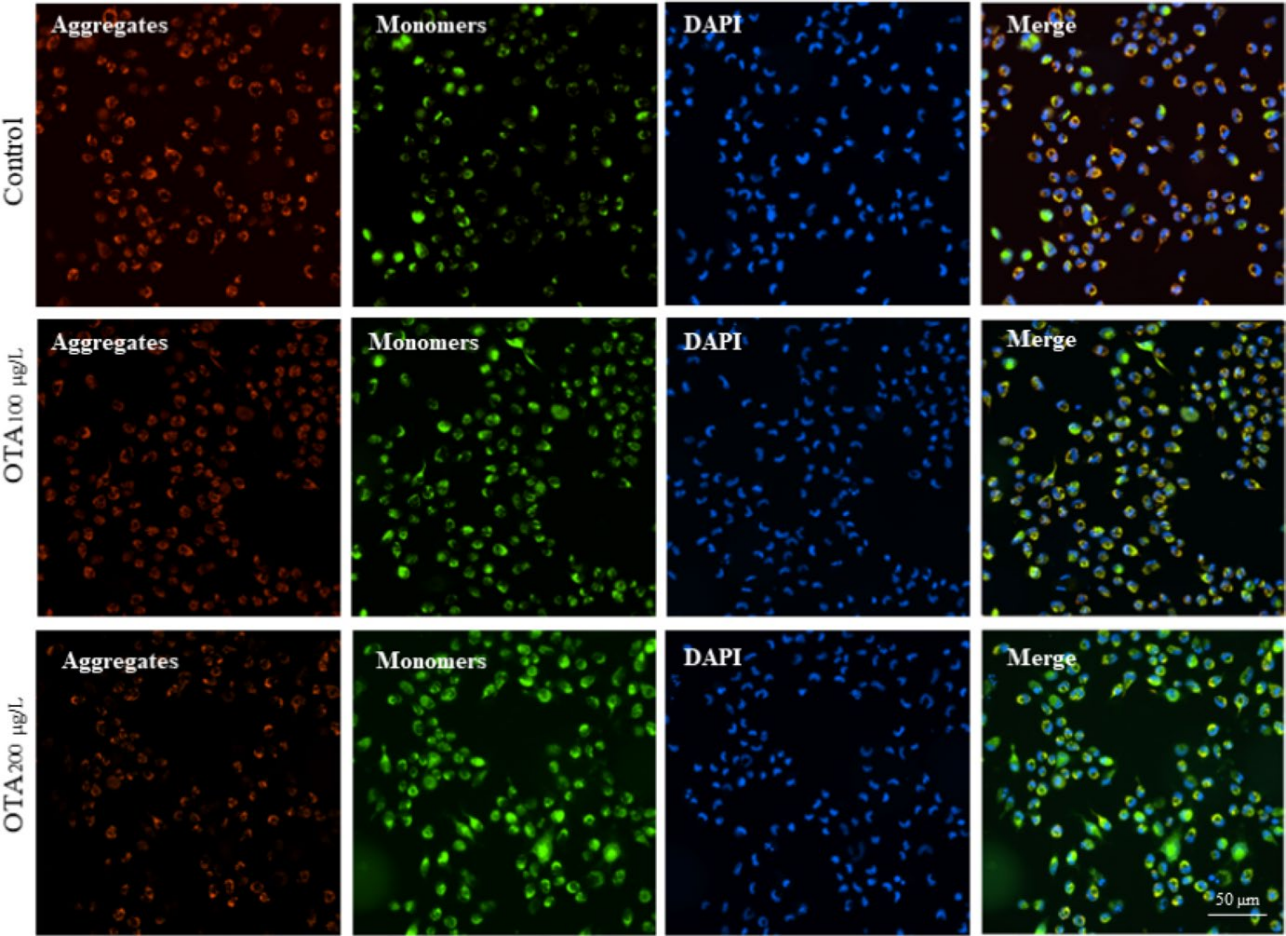
The mitochondrial membrane potential of RGC-5 cells exposed to OTA. Red and green fluorescence represented the aggregate and monomeric form of JC-1, respectively

By utilizing fluorescence microscopy, we conducted a comparison of the changes in mitochondrial membrane potential between RGC-5 cells exposed to OTA for 3 days and normal cells. In normal mitochondria, JC-1 aggregates in the mitochondrial matrix to form a polymer, which emits red fluorescence. When the mitochondrial membrane potential is low, JC-1 cannot aggregate in the matrix of mitochondria and produce green fluorescence. The results demonstrated a significant decrease in mitochondrial membrane potential in the OTA-exposed RGC-5 cells when compared to the normal cells. Furthermore, we observed a concentration-dependent decrease in mitochondrial membrane potential in the OTA-treated RGC-5 cells (Fig. 4).

### OTA-induced RGCs loss in mice

**Fig. 5:**
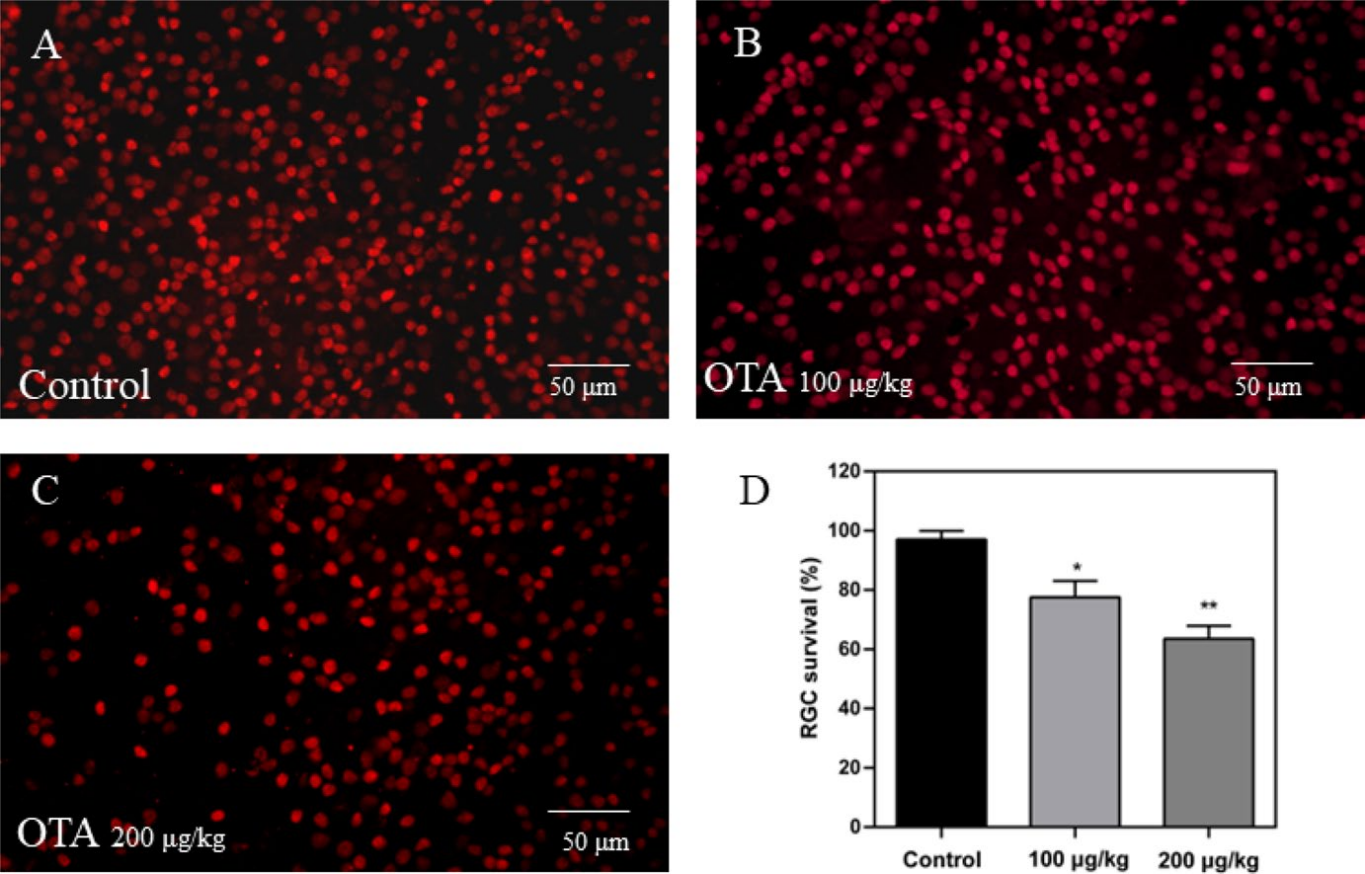
Changes of RGCs after OTA exposure. **a, b** and **c** Retinal flatmounts were stained with Brn3a (red) and observed by a confocal microscope. **d** RGCs survival rates were quantified by ImageJ software (*p < 0.05, **p < 0.01)

To assess the impact of OTA on the RGCs of mice, we proceeded to label the RGCs using Brn3a staining through retinal flat-mount quantification (Fig. 5). Comparing the OTA-exposed group to the control group, we observed a significant reduction in RGCs (*p* < 0.05). The survival of RGCs in the mice exposed to 100 and 200 μg/L OTA decreased by 19.46 and 33.5% compared to control group. To summarize, our results suggested that OTA exposure had a detrimental effect on RGCs in mice.

### OTA induced the visual function impairment of mice

**Fig. 6:**
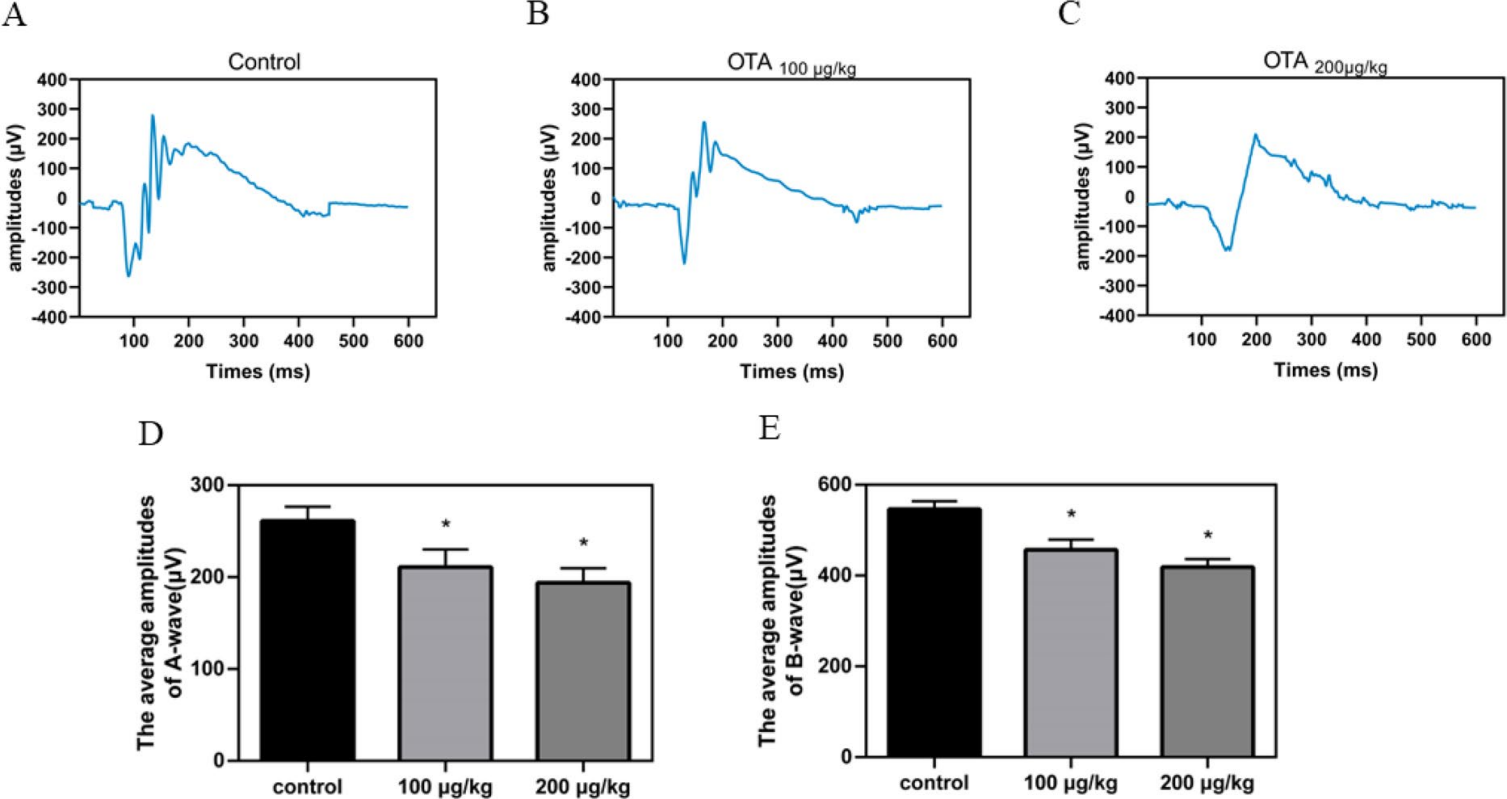
The retinal function was impaired in mice after OTA exposure. **a, b** and **c** Representative traces of flash ERG were recorded at 1.0 cd s/m^2^ in the control group and OTA exposed group. **d** and **e** OTA significantly reduced the amplitudes of both A-wave and B-wave (*p < 0.05)

To investigate the extent of visual function impairment in mice induced by OTA, we utilized scotopic flash electroretinography (ERG) to evaluate changes in retinal function (Fig. 6). Our results revealed significant reductions in the amplitudes of both the A-wave and B-wave in the eyes of OTA-exposed mice compared to normal mice (*p* < 0.05).

## Discussion

The retina is an extremely intricate and complex tissue that plays an essential role in the process of vision. RGCs are a type of neurons within the retina that project and transmit visual information to the brain via the optic nerve. Dysfunction or degeneration of RGCs can significantly impact an individual's visual ability and overall quality of life. This can result in a range of difficulties and challenges in daily activities. OTA is frequently found in foods and has been proven to be a potent neurotoxin. The present study reveals that OTA causes oxidative stress, apoptosis, mitochondrial membrane potential reduction and mitochondrial fragmentation in RGC-5 cells, and induced RGCs loss and retinal function impairment in mice. In the previous study, the report by Van Bergen et al., identifying the RGC-5 cell line as mouse and not rat origin [12]. And Raghu et al. [13] found that RGC-5 cell line was not of RGC origin, but was the cell line 661W, a mouse SV-40 T antigen transformed photoreceptor cell line. Sippl and Tamm [14] reported that RGC-5 may still be a tool in the hands of researchers who want to follow-up in some initial hypotheses that require a transformed retinal cell line of neuronal origin. Therefore, in vitro experiments of RGC-5 cells are not RGC-specific experimental systems.

Oxidative stress under exposure to extreme conditions induces an imbalance between oxidant and antioxidant systems, which could be due to elevated free radical generation and decreased antioxidant activity [15]. Several studies have suggested that both in vitro and in vivo OTA exposure resulted in the overproduction of free radicals and reduction of antioxidant ability [16–18]. Notably, key antioxidant enzymes such as catalase, SOD, and GST are known to play a critical role in safeguarding against oxidative stress [19]. The current study examined the alterations in the levels of both antioxidant enzymes and oxidative stress markers among the groups exposed to OTA. The lower SOD and GST levels, and the higher ROS and MDA levels were observed in the OTA exposed groups. The findings of this study suggest that OTA exposure can lead to oxidative stress, and furthermore, the intensity of oxidative stress was observed to increase with higher concentrations of OTA. Similarly, previous studies have reported that OTA can induce the generation of endogenous ROS and depletion of GSH in rat and pig kidney epithelial cells [20]. And high-concentration OTA exposure was shown to in-crease ROS levels and lead to oxidative DNA damage in both human proximal tubular cell lines [21] and Vero cells [17]. Numerous studies have confirmed that oxidative stress is widely recognized as a potent mediator of apoptosis [22]. In this study, we found that OTA exposure significantly promoted apoptosis of RGC-5 cells. Previous studies have indicated that ROS generated by various sources can accelerate follicular granulosa cells apoptosis [22, 23], and some antioxidant drugs have been confirmed to exert protective effects against follicular granulosa cells apoptosis [24, 25]. Similar research has reported that OTA could induce endoplasmic reticulum stress and ROS production, causing glomerular mesangial cell apoptosis [26]. Hence, we posit that oxidative stress induced by OTA could stimulate apoptosis of RGC-5 cells.

Mitochondria are the primary site within the cell for oxidative phosphorylation and aerobic respiration, and also regulate the production of reactive oxygen species and the occurrence of endogenous cellular apoptosis [27, 28]. Several studies demonstrated that mitochondrial dysfunction could induce oxidative stress, which ultimately resulted in cellular apoptosis [29, 30]. Chayma et al. reported that oxidative stress and apoptosis induced by OTA in human hepatoma cells might be explained because of a loss in the mitochondrial membrane potential [31]. Our study demonstrated that OTA exposure significantly reduced mitochondrial membrane potential in RGC-5 cells. A similar study has reported that H_2_O_2_ can cause a reduction in mitochondrial membrane potential and trigger apoptosis in RGC-5 cells [32]. Mitochondria are highly dynamic organelles, and their continuous cycles of fusion and fission are essential for maintaining critical cellular functions and can impact important physiological and biochemical processes in the body [33]. Fusion results in elongation of the mitochondrial membrane structure, transferring various active substances from smaller mitochondria to larger ones, enhancing mitochondrial function and strengthening oxidative phosphorylation, maintaining stable metabolic pools, and producing sufficient adenosine triphosphate [34]. On the other hand, fission generates small and dysfunctional mitochondria, which often cause mitochondrial dysfunction and lead to a series of diseases [28]. Cummins et al. revealed that changes in mitochondrial ultrastructure and function were observed in secondary degeneration induced by optic nerve damage [35]. In the present study, exposure of RGC-5 cells to OTA resulted in shortened mitochondrial length, decreased tubular mitochondria, and increased spherical mitochondria. These findings suggested that OTA might cause fragmentation of mitochondria in RGC-5 cells, leading to mitochondrial dysfunction.

RGCs are a type of nerve cells located in the retina of the eye. Their primary function is to transmit visual information from the retina to the brain. RGCs are widely studied to gain insights into the degenerative processes that occur in the central nervous system (CNS) following injury-induced neuronal death [36]. The loss of RGCs can have significant consequences on vision and overall visual function. Immunodetection of Brn3a has been established as a reliable method for identifying and quantifying the complete population of rat RGCs in both normal retinas and following various insults [36, 37]. In vivo, the findings of this study, using Brn3a labeling, demonstrated a significant reduction in the survival of RGCs in mice exposed to OTA when compared to the control group. Previous reports have indicated that both A-wave and B-wave amplitudes, recorded by ERG, were observed to be reduced following retinal damage. Results from the current study also showed that the amplitudes of both A-wave and B-wave were reduced following OTA exposure in mice. The decreased survival of RGCs and the impaired retinal function observed in ERG recordings indicate that OTA has retinal toxicity.

## Conclusions

In summary, this study is the first, to our knowledge, to report that OTA can cause damage of retinal function in mice. We found that OTA caused oxidative stress and apoptosis in RGC-5 cells, which might be associated with mitochondrial membrane potential reduction and mitochondrial fragmentation induced by OTA. And in vivo experiments showed that OTA caused severe RGCs death and retinal impairment in mice. This study contributes to a better understanding of the impact of OTA on eye health and provides important clues for the development of preventive and therapeutic methods for RGCs death.

## Data Availability

The data presented in this study are available on request from the corresponding author.
